# Prevalence, Antibiogram, and Multidrug-Resistant Profile of *E. coli* O157: H7 in Retail Raw Beef in Addis Ababa, Ethiopia

**DOI:** 10.3389/fvets.2022.734896

**Published:** 2022-02-24

**Authors:** Aklilu Feleke Haile, Silvia Alonso, Nega Berhe, Tizeta Bekele Atoma, Prosper N. Boyaka, Delia Grace

**Affiliations:** ^1^Aklilu Lemma Institute of Pathobiology, Addis Ababa University, Addis Ababa, Ethiopia; ^2^International Livestock Research Institute, Addis Ababa, Ethiopia; ^3^Ethiopian Public Health Institute, Addis Ababa, Ethiopia; ^4^Department of Veterinary Biosciences, The Ohio State University, Columbus, OH, United States; ^5^Department Microbial Immunity and Infection, The Ohio State University, Columbus, OH, United States; ^6^Infection Diseases Institute, The Ohio State University, Columbus, OH, United States; ^7^International Livestock Research Institute, Nairobi, Kenya; ^8^Natural Resources Institute, Chatham, United Kingdom

**Keywords:** Addis Ababa, antimicrobial, beef, *Escherichia coli* O157:H7, prevalence

## Abstract

*Escherichia coli* O157:H7 is an emerging foodborne pathogen of public health importance. The objectives of this study were to estimate the prevalence and evaluate the antimicrobial susceptibility pattern and multidrug-resistant profile of *E. coli* O157:H7 isolated from raw beef sold in butcher shops in Addis Ababa, Ethiopia. A total of 384 raw beef samples were collected from randomly selected butcher shops across the 10 sub-cities of Addis Ababa. *E. coli* O157:H7 was isolated following ISO-16654:2001 standard, and isolates were tested for resistance to 13 antimicrobial agents using the Kirby–Bauer disk diffusion method. Out of the 384 retail raw beef samples examined, 14 (3.64%) (95% CI = 1.77–5.51%) carried *E. coli* O157:H7 serotype. Of the 14 *E. coli* O157:H7 isolates, 8 (57.14%) were found to be resistant to three or more antimicrobial categories. The frequency of resistant phenotype was more common for ampicillin (92.8%), nitrofurantoin (92.8%), and tetracycline (50%). Multidrug-resistant *E. coli* O157:H7 were present in raw beef sold in butcher shops in Addis Ababa. Thus, more stringent monitoring of antimicrobial use in both human and animal populations should be implemented. In addition, further studies should be conducted to understand the *E. coli* O157:H7 points of contamination and define appropriate risk mitigation strategies.

## Introduction

*Escherichia coli* O157:H7 is an emerging bacterial zoonotic foodborne pathogen of global significance for which cattle is the primary reservoir (1). Cattle shed the bacteria into the environment in their faces, which are then transmitted to humans primarily through the consumption of contaminated raw or undercooked meat (2, 3). The contamination of cattle carcasses or beef can occur during processing and manipulation, such as skinning, evisceration in slaughterhouse, and distribution to butcher shops (4).

While cattle that carry *E. coli* O157:H7 are asymptomatic, infected humans show clinical manifestations ranging from asymptomatic (carrier state) to serious illness. The bacteria adhere to the gut wall of infected people and cause hemorrhagic colitis. Besides, the pathogen also produces toxins that can cause life-threatening complications including hemolytic uremic syndrome (HUS) and thrombotic thrombocytopenic purpura (5, 6).

Early antimicrobial treatment can prevent Shiga toxin-producing *E. coli* O157:H7 infection progression to the HUS (7–9). Studies have shown a significant increase in antimicrobial resistance in *E. coli* O157:H7 (8). This in part may be related to the overuse and misuse of antibiotics in people and food animals (10). In Ethiopia, studies have been confirmed that *E. coli* O157:H7 have developed different percentages of resistance against various commonly used antimicrobial drugs including ampicillin, cephalothin, streptomycin, tetracycline, trimethoprim, amikacin, amoxicillin-clavulanic acid, ciprofloxacin, nalidixic acid, streptomycin, chloramphenicol, nitrofurantoin, and erythromycin (11–18).

Ethiopian food culture includes eating raw beef “Kurt” or minced raw beef “Kitfo,” which increases people's exposure to pathogens. Despite the risk of exposure to *E. coli* O157:H7, limited studies on the magnitude of contamination and risk of *E. coli* O157:H7 and antimicrobial susceptibility has been reported, particularly from developing countries including Ethiopia (19). Such studies can provide valuable information to help in the implementation of strategies to minimize contamination levels.

Earlier studies have reported the occurrence of *E. coli* O157:H7 on raw beef from butcher shops in Ethiopia with results in the range of 0.8–21.9% (11, 12, 14–16). However, the previous studies tend to suffer from small samples and sampling approaches that fail to obtain a representative sample of a population of interest.

Therefore, this study was designed to estimate the prevalence and evaluate the antimicrobial susceptibility pattern and multidrug-resistant profile of *E. coli* O157:H7 isolated from raw beef sold in butcher shops in Addis Ababa, Ethiopia.

## Materials and Methods

### Study Area

**Figure 1:**
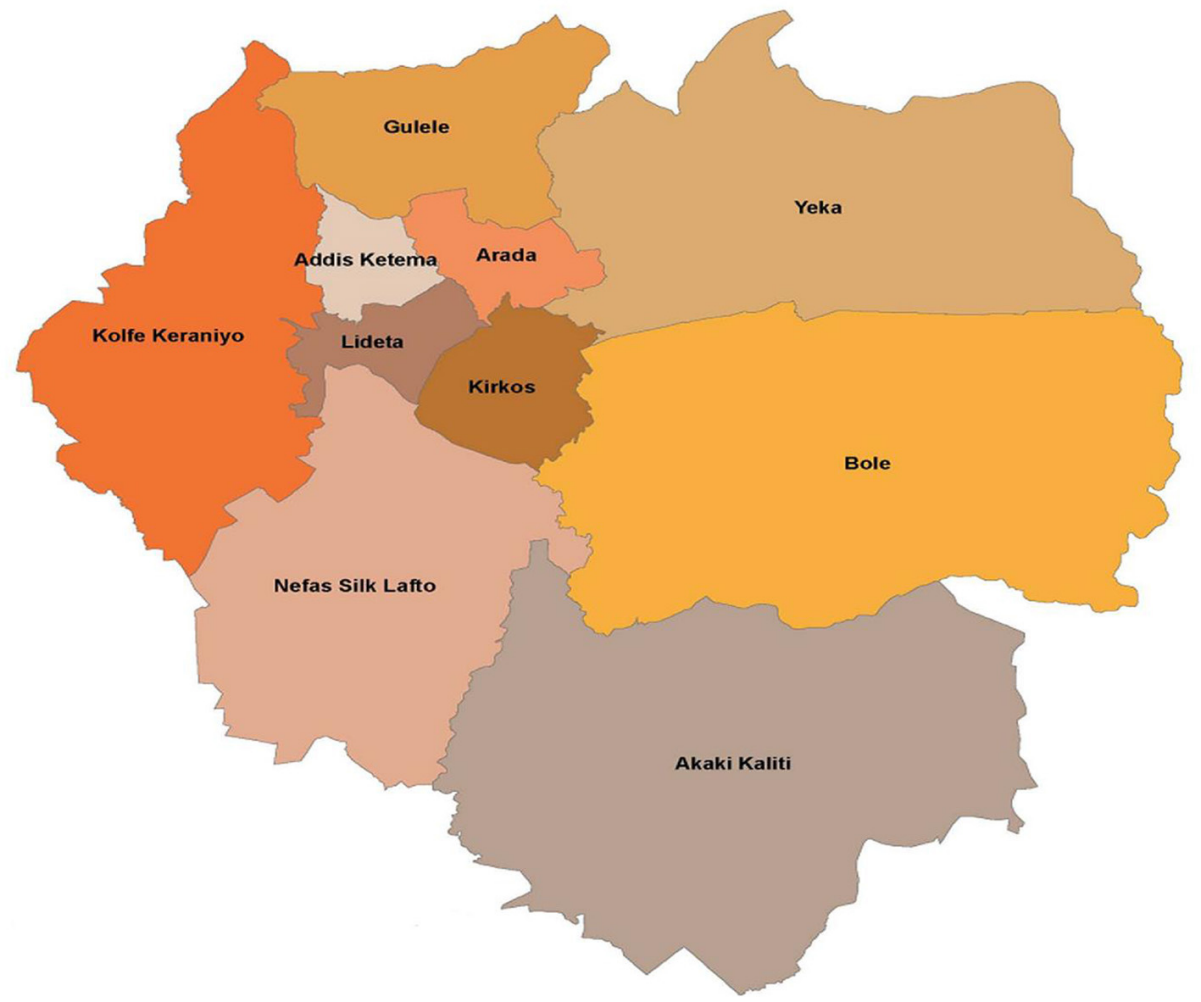
Sub-cities in Addis Ababa included in the study (20).

The study was carried out in Addis Ababa, the capital city of Ethiopia. The city covers 540 km^2^ and is divided into 10 sub-cities (Figure 1). The city lies at an elevation of 2,355 m above sea level and is located at 9°1′48″N 38°44′24″E. The city has minimum, maximum, and average temperatures of 14, 21 and 17.5°C, respectively. The capital city has an estimated human population of 3.15 million.

### Study Design and Sample Size Determination

A cross-sectional study was conducted from October 2018 to December 2019 to determine the prevalence and antimicrobial susceptibility pattern and multidrug-resistant profile of *E. coli* O157:H7 serotypes in retail raw beef samples obtained from butchery shops, in Addis Ababa, the capital city of Ethiopia.

The sample size required was calculated according to Thrusfield (21), from an expected pooled prevalence of 6.5 for the butcher shops (11, 12, 14–16) with a defined precision of 5% and a level of confidence of 95%.


(1) $$\begin{array}{l} \text{n}={\text{Z}}^{2}{\text{P}}_{\text{exp}}\left(1-{\text{P}}_{\text{e}}\text{xp}\right)/{\text{d}}^{2} \end{array}$$

where Z = z statistic for level of confidence; *n* = required sample size; P_exp_ = expected prevalence and a desired absolute precision (d) of 0.05, Z = 1.96. Therefore, the minimum sample sizes were 49 butcher shops. However, in order to increase the precision of the study, a total of 384 butcher shops were included.

### Study Samples and Sampling Methods

The study samples were retail raw beef. A list of active and legally registered butcher shops within the 10 sub-cites and their distribution lines were obtained from Addis Ababa Abattoir Enterprise. A total of 384 butcher shops were selected using the simple random sampling method, and the butcher shops were visited only once A raw beef sample was purchased from each of the randomly selected butcher shops as it was sold to the consumer.

Each sample was placed in a sterile individual plastic bag. The sample was identified by its exclusive sample identification number, which was written on the plastic bag, alongside the sub-city and the date of sampling. Finally, the sample was transported to the Microbiology Laboratory of the Aklilu Lemma Institute of Pathobiology, Addis Ababa University, at cold temperature in a cool box. Upon arrival to the laboratory, the samples were stored in a refrigerator at ±4°C. The samples were processed within 6–12 h from arrival. The detection of *E. coli* O157:H7 was administered consistent with the protocol of ISO-16654:2001 standard (11).

### Sample Preparation and Enrichment

Twenty-five grams of raw beef was weighed and cut into smaller pieces with a sterile scalpel blade on a sterile plate and put in a sterile Stomacher bag. Then, 225 ml of modified Tryptone Soya Broth (TSB) supplemented with Novobiocin (mTSB+N) (1:9) was added to the raw beef and homogenized (Stomacher 400; Seward Medical, Worthing, United Kingdom) at high speed for 2 min. The enrichment sample was then incubated aerobically at 41.5°C for 24 h.

### Isolation

All enriched broths were plated on to cefixime tellurite sorbitol MacConkey agar (CT-SMAC) (Oxoid, Basingstoke, England), supplemented with 0.05 mg/L cefixime and 2.5 mg/L tellurite (Oxoid, Basingstoke, England) (CT-SMAC) (Oxoid, Basingstoke, England) and incubated at 37°C for 24 h. After the incubation period, the CT-SMAC agar plates were examined for the presence of non-sorbitol fermenter colorless colonies, and subsequently, they were sub-cultured on Rainbow agar O157 (Hayward, Berkeley Heights, NJ, USA). The plates were then incubated for 20–24 h at 37°C and observed for the presence of typical black or gray coloration on Rainbow agar O157, indicating pure colonies (22).

### Biochemical Confirmation

Five typical colonies from each Rainbow agar O157 plate were sub-cultured on nutrient agar (Oxoid, Basingstoke, England) for biochemical confirmation by indole formation. The agar plates were incubated at 37°C for 18–24 h. One colony from the pure culture on nutrient agar was inoculated into a tube of tryptone/tryptophan medium (Oxoid, Basingstoke, England) and incubated at 37°C for 24 h. Then, 1 ml of Kovac's reagent (Oxoid, Basingstoke, England) was added and the tube allowed to stand at room temperature for 10 min. The formation of red color indicates a positive reaction (11).

### Serological Identification of O157 and H7 Antigens

Indole-positive colonies were examined for their serological reaction with antiserum to *E. coli* O157:H7 using *RIM E. coli* O157:H7 latex test (Oxoid, Basingstoke, England). Indole-positive colonies were sub-cultured from the nutrient agar to the sorbitol MacConkey agar (Oxoid, Basingstoke, England). For every isolate to be tested, one drop of test latex was dispensed into a well of the test slide. In like manner, one drop of *E. coli* control latex was dispensed into a separate well of the test slide. Using a plastic stick, a portion of the non-sorbitol fermenting colony (NSFC) was removed from the sorbitol MacConkey agar (SMAC) (Oxoid, Basingstoke, England) plate and emulsified in *E. coli* O157 test latex on the slide and spread over the reaction area. Using a fresh plastic stick, the process was repeated with the remaining NSFC and emulsified in *E. coli* control, latex on the slide. The slide was rotated using circular motions for up to 1 min or until agglutination appears. For *E. coli* O157 positives that agglutination occurs with the *E. coli* O157 test latex and the control latex is negative, the isolate was streaked from sorbitol MacConkey agar (Oxoid, Basingstoke, England) to a blood agar (Oxoid, Basingstoke, England) plate and incubated at 37°C for 18–24 h. After 18–24 h incubation, the sweep of growth from the blood agar plate was emulsified in a drop of *E. coli* H7 test latex. Colonies giving an agglutination reaction were confirmed as *E. coil* O157:H7 positive.

### Antimicrobial Susceptibility Testing

The antimicrobial susceptibility was performed, following the standard agar disk diffusion method consistent with CLSI (23) using commercial antimicrobial disks (Table 1). The antimicrobial agents were selected based on the use of antimicrobial agents in the ruminants, potential public health importance, and recommendations from the guideline of antimicrobial susceptibility testing from the Clinical and Laboratory Standards Institute (23).

**Table 1:** Antibiotic disks used to test *E. coli* O157:H7 and their respective concentrations.

**No**.	**Antibiotic disks**	**Disk code**	**Concentration**	**Diameter of zone of inhibition in millimeters (mm)**		
				**Resistant ≤**	**Intermediate**	**Susceptible ≥**
1	Ampicillin	AM	10 μg	13	14–16	17
2	Amoxycillin-clavulanic acid	AMC	20/10 μg	13	14–17	18
3	Amikacin	AK	30 μg	14	15–16	17
4	Ciprofloxacin	CIP	5 μg	15	16–20	21
5	Ceftriaxone	CRO	30 μg	19	20–22	23
6	Cefoxitin	FOX	30 μg	14	15–17	18
7	Nitrofurantoin	F/M	50 μg	14	15–16	17
8	Kanamycin	K	30 μg	13	14–17	18
9	Nalidixic acid	NA	30 μg	13	14–18	19
10	Sulfamethoxazole- trimethoprim	SXT	25 μg	10	11–15	16
11	Tetracycline	TE	30 μg	11	12–14	15
12	Streptomycin	S	10 μg	11	12–14	15
13	Gentamicin	GM	10 μg	12	13–14	15

Each isolated bacterial colony from pure fresh culture was transferred into a tube of 5 ml TSB (Oxoid, Basingstoke, England) and incubated at 37°C for 6 h. The turbidity of the culture broth was adjusted using sterile saline solution or added more colonies to get turbidity comparable with that of 0.5 McFarland standards. The diluted bacterial suspensions were swabbed in three directions uniformly on the surface of Mueller–Hinton agar plates using sterile cotton swabs. After the plates were dried (about 10 min), with the aid of sterile forceps, antibiotic-impregnated disks were placed to the surface of the inoculated plates. Then, the plates were incubated aerobically at 37°C for 24 h. Finally, the diameter of the inhibition zone formed around each disk was measured on a black surface using a transparent ruler by placing it over the plates. The results were classified as sensitive, intermediate, and resistant according to the CLSI (23). *E. coli* (ATCC 25922)-type strains were used as a positive control.

### Multidrug Resistance (MDR)

Multidrug resistance (MDR) was defined as a resistance of a bacterial strain for at least one agent in three or more antimicrobial categories (24).

### Ethical Consideration

The study protocol was ethically approved by the Institutional Review Board of Aklilu Lemma Institute of Pathobiology, Addis Ababa University (Minutes Ref NO: ALIPB IRB/006/2011/2018).

### Data Management and Analysis

The data were entered and coded in MS Excel and then analyzed using IBM SPSS version 25.0 (25). The prevalence was determined by dividing the number of positive samples by the total number of samples examined. Descriptive statistics such as frequency and percentages were used to describe the proportion of resistant, intermediate, or susceptible strains. The difference in prevalence by sub-city was determined using the chi-square (χ^2^) test. A *p*-value < 0.05 was considered indicative of a statistically significant difference.

## Results

### Prevalence

Out of 384 raw beef samples examined, 14 (3.64%) (95% CI = 1.77–5.51%) were positive to *E. coli* O157:H7 serotypes.

*E. coli* O157:H7 serotypes were detected in Addis Ketema (2%), AkakiKality (1.47%), Arada (14.29%), Bole (4.65%), Gullele (7.14%), Kirkos (69%), KolfeKeraneo (0%), Lideta (6.9%), Nefassilk (1.72%), and Yeka (4.76%). Variation in the prevalence between the butcher shops from the different sub-cities was not statistically significant (*p* > 0.05) (Table 2).

**Table 2:** Prevalence of *E. coli* O157:H7 by risk factor.

**Risk factor**		**Number examined**	**Positive no. (%)**	***X* ^2^**	***df***	***p*-value**
Sub-city	Addis Ketema	50	1 (2)	13.039	9	0.161
	Akaki Kality	68	1 (1.47)			
	Arada	21	3 (14.29)			
	Bole	43	2 (4.65)			
	Gullele	14	1 (7.14)			
	Kirkos	29	2 (6.9)			
	Kolfe Keraneo	51	0 (0)			
	Lideta	29	2 (6.9)			
	Nefassilk	58	1 (1.72)			
	Yeka	21	1 (4.76)			

### Antimicrobial Susceptibility Pattern

The result of the antimicrobial susceptibility test of the14 *E. coli* O157:H7 serotypes isolated from raw beef samples with 13 selected antimicrobial agents is shown in Table 3.

**Table 3:** Antimicrobial susceptibility pattern of *E. coli* O157:H7 isolates (*n* = 14).

**Antimicrobial used**	**Sensitive no. (%)**	**Intermediate no. (%)**	**Resistant no. (%)**
Ampicillin (AM)	1 (7.14)	0 (0)	13 (92.8)
Amoxicillin-clavulanate (AMC)	9 (64.2)	2 (14.2)	3 (21.4)
Amikacin (AK)	14 (100)	0 (0)	0 (0)
Ciprofloxacin (CIP)	14 (100)	0 (0)	0 (0)
Ceftriaxone (CRO)	14 (100)	0 (0)	0 (0)
Cefoxitin (FOX)	11 (78.5)	2 (14.2)	1 (7.14)
Nitrofurantoin (F/M)	1 (7.14)	0 (0)	13 (92.8)
Kanamycin (K)	10 (71.4)	4 (28.5)	0 (0)
Nalidixic acid (NA)	13 (92.8)	1 (7.14)	0 (0)
Sulfamethoxazole trimethoprim (SXT)	13 (92.8)	0 (0)	1 (7.14)
Tetracycline (TE)	5 (35.7)	2 (14.2)	7 (50.0)
Streptomycin (S)	4 (28.5)	8 (57.1)	2 (14.2)
Gentamicin (GM)	12 (85.7)	2 (14.2)	0 (0)

All the 14 *E. coli* O157:H7 serotypes' isolates from raw beef were found to be susceptible to amikacin (100%), ciprofloxacin (100%), and ceftriaxone (100%). Furthermore, the isolates showed high susceptibility to sulfamethoxazole-trimethoprim (92.8%), nalidixic acid (92.8%), gentamicin (85.7%), cefoxitin (78.5%), kanamycin (71.4%), and amoxicillin-clavulanic acid (64.2%). The results of the present study on antimicrobial sensitivity test indicated high resistance to ampicillin (92.8%), nitrofurantoin (92.8), and tetracycline (50.0%).

### Multidrug Resistance Profiles

Out of the 14 *E. coli* O157:H7 isolates, 8 (57.14%) were found to be resistant to three or more antimicrobial categories. MDR profiles against three, four, and five antimicrobial categories were resistant to 5 (35.7%), 1 (7.1%), and 2 (14.3%), respectively. The frequency of resistant phenotype was more common for ampicillin, nitrofurantoin, and tetracycline (Table 4).

**Table 4:** MDR profile of *E. coli* O157:H7 isolates.

**Number of antimicrobials**	**Antimicrobials**	**No. of isolates (%)**
Three	AM,F/M,TE	3 (21.4)
	AM, F/M, AMC	1 (7.14)
	AM, F/M, S	1 (7.14)
Four	AM, F/M, AMC, TE	1 (7.14)
Five	AM, F/M, AMC, FOX, TE	1 (7.14)
	AM, F/M, S, SXT, TE	1 (7.14)
	Total MDR	8 (57.14)

*AM, ampicillin; AMC, amoxicillin-clavulanate; FOX, cefoxitin; F/M, nitrofurantoin; S, streptomycin; SXT, sulfamethoxazole + trimethoprim; TE, tetracycline*.

## Discussion

Foodborne infections are major health concerns in developing countries including Ethiopia. The surveillance and monitoring of foodborne pathogens provide crucial information on planning, implementing, and evaluating food safety systems. Therefore, appropriate information on the contamination level and antimicrobial susceptibility of *E. coli* O157:H7 in retail raw beef may have implications in strengthening the surveillance system of foodborne diseases as well as is important to design prevention and control measures to decrease the risk of contamination. The prevalence of *E. coli* O157:H7 found in raw beef samples in the present study was 14/384 (3.64%) (95% CI = 1.77–5.51%). Similar to our findings, *E. coli* O157:H7 was identified in 1/25 (4%), 1/25 (4%), and 1/30 (3.3%) of raw beef samples at butcher shops in Addis Ababa, Batu, and Holetta, respectively (16). In contrast to our findings, the lower prevalence in raw beef samples was 3/150 (2%) in Hawassa and 1/125 (0.8%) in Addis Ababa and Debre Berhan (14, 15). Higher prevalence was described in butcher shops in Bishoftu 8/86 (9.3%) and 2/30 (6.7 %) and in Addis Ababa 14/64 (21.9%) (11, 12, 16). The variation of these findings might depend on different factors, e.g., abattoir, butcher conditions, sample size, and laboratory methods.

In this study, no statistically significant variation in the prevalence rate among the sub-cities butcher shops of beef samples (*p* > 0.05) was observed. This might be due to butcher shops sourcing their cattle carcasses from the main abattoir in the city. The small number of positives also means that a much larger sample size would be needed to identify any differences.

All of the 14 isolates of raw beef were susceptible to amikacin, ciprofloxacin, and ceftriaxone. Furthermore, the isolates showed high susceptibility to sulfamethoxazole-trimethoprim, nalidixic acid, gentamicin, cefoxitin, kanamycin, and amoxicillin-clavulanic acid. Similar findings have been reported by other researchers from Ethiopia (11, 12, 14–16, 18, 26). The *E. coli* O157:H7 strains isolated from raw beef had high resistance to ampicillin (92.8%), nitrofurantoin (92.8%), and tetracycline (50.0%). Similarly, studies from Ethiopia (13–16, 18, 26, 27) and Nigeria (27) revealed high resistance among *E. coli* O157:H7 isolates to ampicillin. However, 90% susceptibility of ampicillin was reported in Bishoftu (11). Nitrofurantoin resistance was reported in Somalia and Hawassa (13, 15). Drugs like ampicillin and nitrofurantoin have long been used for the management of various infections in Ethiopia, and high rate of resistance to these drugs might have developed as a consequence of this prolonged use (28).

Moreover, the findings of antimicrobial susceptibility test showed that 50% of *E. coli* O157:H7 isolates from raw beef resistance to tetracycline (14, 17). This is in agreement with previous studies from Ethiopia (11, 13, 26, 27) and Nigeria (27). This might be related to the broad use of tetracycline in the management of various infections in the livestock in Ethiopia (29).

Among the 14 *E. coli* O157:H7 isolates from raw beef tested, 8 (57.14%) were resistant to three or more classes of antibiotics. The occurrences of multidrug-resistant isolates (17.9–92.5%) were also reported in previous studies in Ethiopia (11–13, 17, 18). The occurrence of MDR may be associated with indiscriminate utilization of antimicrobial agents, which was not elucidated with the current study method. Furthermore, the transmission of MDR bacteria *via* the consumption of meat have been propounded as a potential source in Africa (30, 31).

The present study had some limitations. The use of immunomagnetic separation (IMS) with enrichment in broth culture enhances the isolation of *E. coli* O157 from samples with a low concentration of bacteria (32). In this study, enrichment without IMS was employed to isolate *E coli* O157:H7. Nevertheless, the present study revealed that multidrug-resistant *E. coli* O157:H7 were present in raw beef sold in butcher shops in Addis Ababa, Ethiopia. Given the low infective dose of *E. coli* O157:H7 [10 colony forming unit (CFU)/g] and the cultural habit of eating raw beef in the society, the current prevalence should be considered important from a public health standpoint. These findings should be communicated with government and projects working with butchers along with the information on reducing the risk. Thus, more stringent monitoring of antimicrobial use in both human and animal populations should be implemented. In addition, further studies should be conducted to understand the *E. coli* O157:H7 points of contamination and define appropriate risk mitigation strategies.

## Data Availability Statement

The raw data supporting the conclusions of this article will be made available by the authors, without undue reservation.

## Author Contributions

AH: conceived and designed the study, conducted the study, analyzed the data, and wrote the paper. SA: conceived the study, provided guidance to its design, and reviewed the manuscript. NB: designed the study, analyzed the data, and wrote the paper. TA: conducted the study and analyzed the data. PB: reviewed the paper and wrote the paper. DG: conceived and designed study, analyzed the data, and reviewed the manuscript. All authors contributed to the article and approved the submitted version.

## Funding

This study was conducted under the project of the International Livestock Research Institute (ILRI), funded by CGIAR Research Program on Agriculture for Nutrition and Health.

## Conflict of Interest

The authors declare that the research was conducted in the absence of any commercial or financial relationships that could be construed as a potential conflict of interest.

## Publisher's Note

All claims expressed in this article are solely those of the authors and do not necessarily represent those of their affiliated organizations, or those of the publisher, the editors and the reviewers. Any product that may be evaluated in this article, or claim that may be made by its manufacturer, is not guaranteed or endorsed by the publisher.
